# Microbial dynamics across tri-trophic systems: insights from plant–herbivore–predator interactions

**DOI:** 10.1093/femsec/fiaf065

**Published:** 2025-06-18

**Authors:** Hong Yan, Endong Wang, Guo-Shu Wei, Xuenong Xu, Mark R H Hurst, Bo Zhang

**Affiliations:** College of Plant Protection, Hebei Agricultural University, Baoding 071000, Hebei Province, China; State Key Laboratory for Biology of Plant Diseases and Insect Pests, Institute of Plant Protection, Chinese Academy of Agricultural Sciences, Beijing 100193, China; State Key Laboratory for Biology of Plant Diseases and Insect Pests, Institute of Plant Protection, Chinese Academy of Agricultural Sciences, Beijing 100193, China; Key Laboratory of Natural Enemies Insects, Ministry of Agriculture and Rural Affairs, Beijing 100193, China; College of Plant Protection, Hebei Agricultural University, Baoding 071000, Hebei Province, China; State Key Laboratory for Biology of Plant Diseases and Insect Pests, Institute of Plant Protection, Chinese Academy of Agricultural Sciences, Beijing 100193, China; Key Laboratory of Natural Enemies Insects, Ministry of Agriculture and Rural Affairs, Beijing 100193, China; Resilient Agriculture, AgResearch, Lincoln Research Centre, Christchurch 8140, New Zealand; State Key Laboratory for Biology of Plant Diseases and Insect Pests, Institute of Plant Protection, Chinese Academy of Agricultural Sciences, Beijing 100193, China; Key Laboratory of Natural Enemies Insects, Ministry of Agriculture and Rural Affairs, Beijing 100193, China

**Keywords:** bacterial diversity, deterministic processes, fitness, microbiome, predatory mite

## Abstract

Microbes play a critical role in regulating tri-trophic interactions among plants, herbivores, and their natural enemies, influencing key ecological and evolutionary processes. To fully understand these interactions through the food chain, a well-defined tri-trophic system is required. We investigated microbial dynamics involving plants (beans, cucumbers, and eggplants), spider mites (*Tetranychus urticae*), and predatory mites (*Phytoseiulus persimilis*) through 16S rRNA gene sequencing. The results revealed significant variations in microbiota across different trophic levels. Source tracking analysis indicated that microbiota at each trophic level were rarely inherited from the previous one, and deterministic processes played a key role in shaping the endosphere communities of these levels. Most shared zero-radius operational taxonomic units across each trophic level belonged to *Pseudomonas, Bacillus*, and *Staphylococcus*. Leaf microbiota differed among plants, while spider mites harbored similar microbiota. Notably, the microbiota of predatory mites on eggplants differed significantly from those on the other two plants. Biomarker selection and correlation analyses revealed that the abundance of *Methylobacterium* and *Stenotrophomonas* was strongly correlated with the improved fitness of predatory mites across different plants. Our study highlights the complex and dynamic nature of microbial communities across different trophic levels in a well-defined plant–herbivore–predator system.

## Teaser

16S rRNA gene sequencing of a plant–herbivore–predator system reveals distinct microbiota across trophic levels, limited vertical transmission, and *Methylobacterium*/*Stenotrophomonas* linked to predator fitness.

## Introduction

Tri-trophic interactions among plants, herbivores, and natural enemies play a vital role in shaping ecosystems and driving key ecological and evolutionary processes (Tao et al. 2017). Microorganisms, are often hidden players integral to these multitrophic systems. The complex interplay between trophic relationships and environmental factors shapes the composition of microbial communities at each trophic level. In turn, microbial changes can profoundly influence the dynamics within tri-trophic interactions (Frago et al. 2012, Kim et al. 2019). For example, the microbiota of arthropod herbivores, which is shaped by both the host genotype and plant species, impacts not only the herbivores themselves but also the behavior of their predators (Brady et al. 2014, Ushio et al. 2015). Recently, there has been a surge in research on microbiota in plant–herbivore systems, including studies on how rhizobacteria influence plant traits and herbivore performance (Schädler and Ballhorn 2016, Rasmann et al. 2017, Molinari and Leonetti 2023) and how insect microbiota affects ecological communities by altering interactions with plants and competitors (Frago et al. 2012, Frago and Zytynska 2023). However, the interaction with the third trophic level remains unexplored.

Understanding the mechanisms of microbial transmission between plants and insects across trophic levels has become clearer with a growing emphasis on multitrophic linkages. Microbes can transfer from host plants to insects, providing beneficial effects (Berasategui et al. 2022). Additionally, microbes also migrate from insects to host plants and undergo horizontal transmission among insect populations (Chrostek et al. 2017, Li et al. 2017, Gu et al. 2023). Host microbiota can be acquired from species across different trophic levels, as demonstrated by many insects acquiring microbiomes from the soil rather than the host plant (Kikuchi et al. 2012, Hannula et al. 2019, Gomes et al. 2020, Zhang et al. 2022). In specific tri-trophic systems, such as the interaction between plants (downy and holm oaks), caterpillars, and avian blue tits, it has been demonstrated that hosts from adjacent trophic levels share more similar bacterial microbiota compared to those separated by two trophic levels, further supporting the possibility of microbial transfer (Dion-Phénix et al. 2021). These intricate relationships within the tri-trophic system require a holistic evaluation from an integrated perspective that encompasses all trophic levels.

The tri-trophic interactions between plants, spider mite *Tetranychus urticae* and predatory mite *Phytoseiulus persimilis* have long been established in ecological research, although most studies on this system have primarily focused on the relationships between microorganisms and plant–spider mites or spider mites–predatory mites (Zhu et al. 2019, Yan et al. 2024). As a pest mite feeding on over 1100 plant species (Grbić et al. 2011), host plants have been found to significantly impact the microbial community of *T. urticae* (Zhu et al. 2019). Similarly, *P. persimilis* the specialist predator of *T. urticae*, mostly acquired their bacterial communities from their prey (Yan et al. 2024). However, the transfer of microorganisms within the tri-trophic level and their effects on this system have remained unclear (Pekas et al. 2017). Based on the strict predatory ability of *P. persimilis* against *T. urticae* and previous research on microorganisms of this system, we hypothesize that the third trophic level could be affected by the previous two levels.

Continued advancements in our understanding of microbial dynamics within these tri-trophic systems are used to elucidate the intricate relationships and interactions that drive ecosystem functioning and species coexistence. In this study, isofemale lines of *T. urticae* and *P. persimilis* were assessed on three different plants, including white kidney bean, cucumber, and eggplant to establish respective tri-trophic systems. We utilized 16S rRNA gene sequencing to investigate the microbiota flow and dynamics within the endosphere of the roots, leaves, spider mites, and predatory mites. Additionally, we differentiated the distinct bacterial species from the predators in different tri-trophic systems and correlated them to host fitness. By investigating the transfer of microorganisms across different trophic levels, we elucidate the potential mechanism of transfer for the functioning and stability of tri-trophic interactions.

## Materials and methods

### Plants and mites

Cucumber (Family Cucurbitaceae, variety Jinyou 209), and eggplant (Family Solanaceae, variety Brigitte) seedlings were provided by Tian’an Organic Farm (Beijing, China). Bean (Family Fabaceae, variety White kidney bean) seedlings were bought from Beijing Huazhaofeng Trading Co., Ltd. The predatory mites, *P. persimilis*, used in these experiments were maintained on spider mites *T. urticae* feeding on bean leaves in the Laboratory of Predatory Mites, Institute of Plant Protection, Chinese Academy of Agricultural Sciences. All mites were reared at 25 ± 1°C, 70% ± 5% RH and L14: D10 photoperiod in incubators (380 L, MLR-352H-PC, Ningbo, China).

### Establishment of mite isofemale lines for three tri-trophic systems

**Figure 1.**
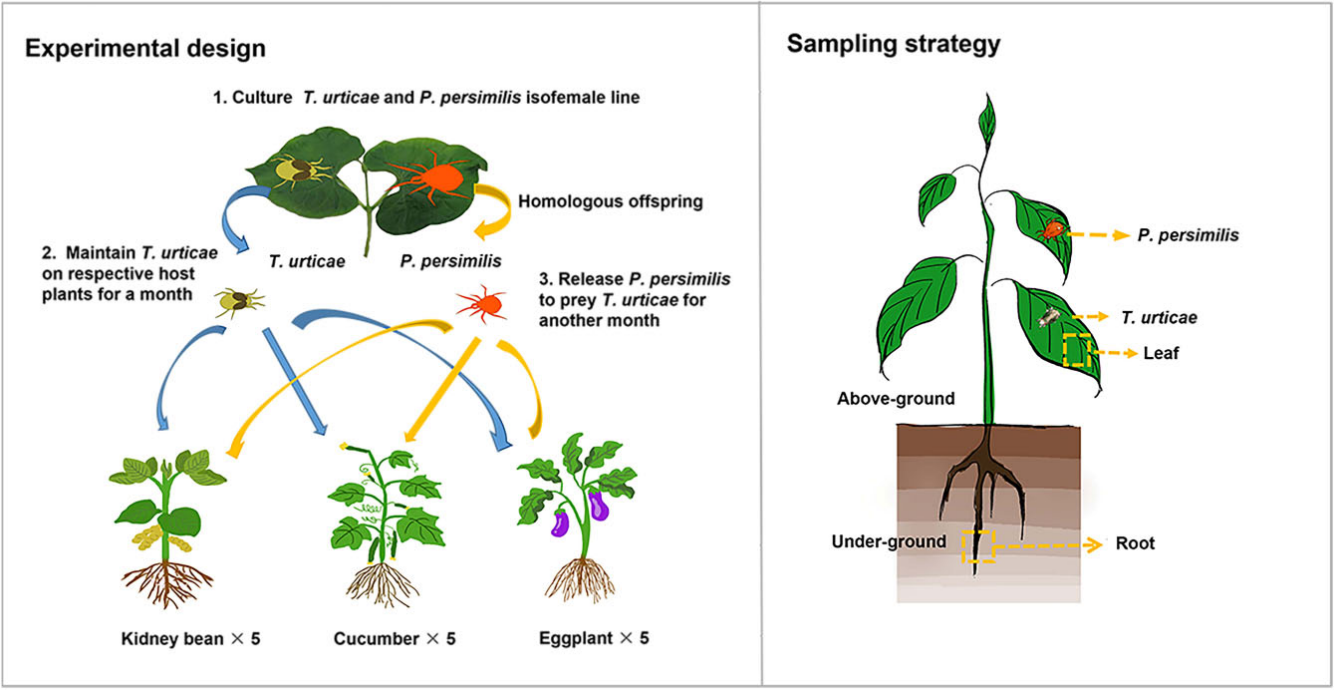
The schematic diagram of sampling, with the left side depicting the experimental procedure and the right side indicating the sampling strategy. Three plant species (bean, cucumber, and eggplant) were placed with spider mites and predatory mites, respectively. Samples of roots, leaves, spider mites, and predatory mites were collected, with five biological replicates per sample type, resulting in a total of 60 samples. DNA was extracted using the DNeasy Plant Mini Kit for plant tissues and the DNeasy Blood & Tissue Kit for mite samples. DNA quality was assessed by Nanodrop (A260/A280) and agarose gel electrophoresis. Sequencing was performed on the Illumina MiSeq platform, and raw FASTQ files were processed using Trimmomatic v0.38 to remove adapters and low-quality reads.

To minimize genetic variation within the mite colonies, we established isofemale lines for both *T. urticae* and *P. persimilis*. A gravid female mite was placed in a clip cage arena to oviposit. The clip cage arena comprises four tightly clipped layers: a transparent acrylic board (30 mm^3^ × 20 mm^3^ × 3 mm^3^) with a 10-mm diameter hole in the middle, a leaf disc and two pieces of rectangular glass (30 mm^3^ × 20 mm^3^ × 1 mm^3^) placed at the top and bottom, that are held together with a metal clip. Once the eggs matured into adults, a pair of male and female were selected for mating over 24 h. By removing the male, the offspring produced within 48 h were removed to a plastic box to establish a stable mite line until commencement of the experiment. Bean, cucumber, and eggplant plants were each grown in six pots (diameter = 30 cm) with peat soil, with five plants per pot under the condition of 25 ± 1°C, 70% ± 5% RH, and L14: D10 photoperiod in a walk-in plant growth chamber. Five pots of plants were used to place *T. urticae* and *P. persimilis* as five biological replicates. After 3 months of plant growth, 200 spider mites (*T. urticae*) were introduced into each pot and allowed to reproduce for 1 month, completing ~3 generations (generation time—10 days). After this time, 30 gravid *P. persimilis* females were released onto the plant leaves per pot, and the pots were left for a month. Through this time *T. urticae* completed a further three generations (totaling six generations over the 2 months), while with a generation time of 5 days *P. persimilis* completed six generations. Under ideal conditions, adult female *P. persimilis* exhibit a daily predation rate of ~12 adult female *T. urticae*, while each adult female *T. urticae* has an oviposition capacity of ~16 eggs per day (Najafabadi et al. 2014, Zhou et al. 2024). The sample collection of spider mites and predatory mites commenced 1 month after the introduction of *P. persimilis* (Fig. 1). The leaves from the sixth pot of each of the three host plants that were not challenged with mites, were used to observe the developmental period and fecundity of *T. urticae*.

### Sample collection, DNA extraction, and 16S rRNA gene amplicon sequencing

For each plant species, the tap root 10 cm length from the base of foliage was sampled and rinsed with sterile water until all visible soil particles were removed. Three leaves were selected from each plant pot, and a 2 cm × 2 cm piece of leaf around the main vein was cut using a scalpel. The mites were meticulously cleaned off under a microscope (SZ650BP, Chongqing) using a size 00 000 paint pen brush. To eliminate surface-derived microbial contamination and ensure exclusive analysis of endosphere-associated communities, the roots and leaves were independently placed in 50 ml centrifuge tubes containing 10 ml of 0.5% sodium hypochlorite. The tubes were then manually shaken by hand for 1 min. This process was repeated twice, from where the samples were repeatedly rinsed three times with sterile water. The samples were transferred to a sterile Petri dish to drain residual water, then cut into small pieces (~5 mm) on the benchtop and stored in 1.5 ml centrifuge tubes at −20°C. For processing, 20 *T. urticae* and 20 *P. persimilis* mites were independently collected from plants (per pot) and the different species transferred into separate 1.5 ml microcentrifuge tubes. The mites were then cleaned using the same procedure as used for the plants. For each plant, samples were collected from the root, leaf, spider mites, and predatory mites, with five replicates for each sample. In total, there are 60 DNA samples across the three plant systems.

The samples were ground using an electric tissue grinder (3000–8000 r/m; Shanghai Sangon Biotech Co., Ltd). DNA extraction from root, leaf, and mite samples was performed using the DNeasy Plant Mini Kit (Juhua Tech Technology Company, Beijing) and the DNeasy Blood & Tissue Kit (Yishan Huitong Technology Company, Beijing), respectively. The V4 region of the bacterial 16S rRNA gene was amplified using the primers 515F (5′-GTGCCAGCMGCCGCGG-3′) and 806R (5′-GGACTACHVGGGTWTCTAAT-3′) with Phusion High-Fidelity DNA Polymerase (M0530S, NEB). The polymerase chain reaction (PCR) protocol included an initial denaturation at 98°C for 30 s, followed by 35 cycles of 98°C for 10 s, 55°C for 20 s, and 72°C for 15 s, with a final extension at 72°C for 5 min (Yan et al. 2021). PCR products were then purified using the Omega Bio-tek E.Z.N.A Cycle-Pure Kit. Sequencing was carried out on the Illumina MiSeq platform. Raw FASTQ files were processed using Trimmomatic version 0.38 (Bolger et al. 2014) and the USEARCH fastq_filter parameter (version 11) to remove low-quality reads (Edgar and Flyvbjerg 2015). Merged sequences were then clustered into Zero-radius operational taxonomic units (ZOTUs) using USEARCH UPARSE (Edgar 2013). Taxonomic classification and prediction were performed using the Ribosomal Database Project (Cole et al. 2014). ZOTUs identify biological sequences with single-nucleotide resolution (100% similarity) and simultaneously removes chimeric sequences and low-abundance reads. Compared to traditional OTU clustering methods (e.g. 97% similarity thresholds) or amplicon sequence variants, ZOTUs utilize a global denoising strategy that more rigorously eliminates sequencing errors, improving the resolution of microbial diversity analyses (Li et al. 2024).

### Mites fitness in three tri-trophic systems

For each plant species to compare the differences in mite performance, 20 adult spider mites from each of the three different plant species were collected using a paint brush (size: 00 000) and placed them onto fresh leaves of the appropriate plant species for egg laying. Approximately 50 eggs sourced from eggs laid within 12 h were placed into clip cage arenas, and the development time recorded from eggs to adult stage. Once they reached adulthood, individuals were paired for mating, and males were removed after 24 h. Subsequently, the daily egg-laying quantity of female mites was recorded for 7 consecutive days. We selected the leaves infected with spider mites from the five pots mentioned above and examined them under a microscope from where any predatory mites were removed. These leaves were then crafted into clip cage arenas and 30 gravid *P. persimilis* from each plant were selected to lay eggs in 10 clip cage arenas (three *P. persimilis* per cage), from where 50 eggs were collected within 12 h. These eggs were placed in clip cage arenas made from leaves infected with spider mites. The mites were then monitored for their developmental stages and fecundity (egg-laying numbers) over the following 7 days.

### Data analysis

Both *α*- and *β*-diversity metrics were calculated using the USEARCH package. To mitigate potential confounding effects of variable sequencing depths across samples, the relative abundance of each ZOTU was calculated by normalizing the total abundance of all ZOTUs in each sample to 1. This was achieved through dividing individual ZOTU abundances by the corresponding sample’s total ZOTU abundance. To assess overall microbiome composition differences among groups, we employed permutational multivariate analysis of variance (PERMANOVA) based on Bray–Curtis distance matrices. Stacked bar plots illustrating the composition of the top 20 order-level taxa across distinct trophic levels were generated using the *ggplot2* and *tidyverse* packages in R. Core ZOTUs (defined as ZOTU abundance ≥0.001 per sample, with at least three replicates per group exceeding 0.001) from different trophic levels on each plant were used to construct a network. The network analyses were conducted in R, calculating Spearman correlation scores and retaining only robust correlations (Spearman’s *r* > 0.6 or *r* < −0.6) that were statistically significant (*P* < .05). The results were visualized using Gephi (Bastian et al. 2009).

We employed SourceTracker (v1.0), a Bayesian-based approach, to estimate the microbial sources for the plants and mite species (Knight and Kelley 2011). To evaluate the relative contributions of stochastic processes to microbial community assembly, the Neutral Community Model (NCM) was implemented to analyze ZOTU occurrence patterns across trophic levels within each plant system. We modeled the observed relative abundance–frequency distributions of microbial taxa using a beta distribution derived from neutral theory. In this framework, the detection probability of a ZOTU depends on its mean relative abundance (m) in the metacommunity and the migration rate (Nm). Model parameters (Nm and *R*²) were estimated via maximum-likelihood estimation using the fit_neutral function from the R package micropower (v2.1.0). Parameter uncertainties were assessed through 1000 bootstrap iterations. Model fit was evaluated using the coefficient of determination (*R*²) between observed and predicted ZOTU detection frequencies. To statistically validate deviations from neutral expectations, we applied the Kolmogorov–Smirnov test (*α* = 0.05) to compare empirical data against model predictions. Community assembly processes were determined by combining *R*² and Nm, where *R*² < 0.5 was recognized as indicative of deterministic processes (Sloan et al. 2006).

To identify biomarkers taxa specific to spider mites and predatory mites on different plants, we used linear discriminant analysis effect size (LEfSe) with a Wilcoxon *P*-value of .05 and LDA > 4/5 (Segata et al. 2011). The analysis was carried out using the LEfSe Galaxy tool. The *corrplot* package in R was used to create heatmap to assess the relationship between these biomarkers and mite fitness. The R packages *VennDiagram, openxlsx, ggvenn*, and *paletteer* to generate the Venn diagram depicting the shared and special core ZOTUs in spider mites and predatory mites across different plants, as well as the shared ZOTUs across each trophic level. Based on the results of Shapiro–Wilk tests for normality, we conducted one-way ANOVA with Turkey’s *post hoc* tests to evaluate the cumulative relative abundance of shared core ZOTUs in spider mites and predatory mites across different plants. A general linear regression analysis was performed using SPSS 22.0 to explore the relationship between the cumulative relative abundance of shared core ZOTUs and mite fitness. The data were visualized using GraphPad Prism 10.2.0.

## Results

### Overview of sequencing results

A total of 4 245 520 reads were obtained from 60 samples, averaging 70 758 reads per sample after quality control. The rarefaction analysis indicated that sufficient sampling depth was achieved for all samples. Based on the threshold of 97% similarity and 0.1% relative abundance, the cluster analysis yielded 7348 ZOTUs belonging to 29 phyla.

### Diversity of bacterial microbiomes across different trophic levels

**Figure 2.**
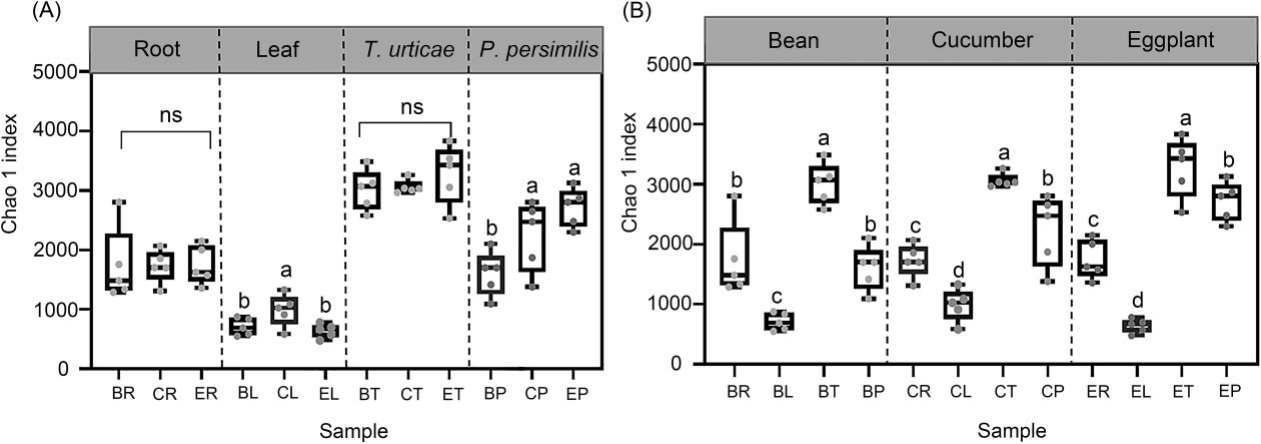
Alpha diversity indices of bacterial communities. (A) The same trophic levels of different plants; (B) various trophic levels of the same plants. BR: the root of bean; CR: the root of cucumber; ER: the root of eggplant; BL: the leaves of bean; CL: the leaves of cucumber; EL: the leaves of eggplant; BT: *T. urticae* on bean, CT: *T. urticae* on cucumber; ET: *T. urticae* on eggplant; BP: *P. persimilis* on bean; CP: *P. persimilis* on cucumber; and EP: *P. persimilis* on eggplant. ns, not significant. Different lower-case letters indicated significant differences among different trophic levels (*P* < .05).

Based on 16S rRNA gene profiling, microbial diversity (Chao 1 value) of the roots of bean, cucumber, and eggplant showed no significant differences (*F*_(2,12)_ = 0.001, *P* = .999, Fig. 2A). However, cucumber leaves exhibited greater microbial diversity compared to beans and eggplants, with a Chao1 value of 989.46 (*F*_(2,12)_ = 4.649, *P* = .032, Fig. 2A). In contrast, there were no significant differences in microbial diversity among spider mites across the three plants (*χ*^2^_(2)_ = 1.580, *P* = .454, Fig. 2A). Notably, the Chao1 value of the predatory mite microbiota on eggplants was 2716, significantly higher than that of predatory mites on beans (*F*_(2,12)_ = 7.775, *P* = .007, Fig. 2A). Across all plant types, Chao1 richness was highest at the second trophic level and lowest in the leaves (Fig. 2B). Similarly, in both cucumber and eggplant systems, spider mites at the second trophic level exhibited the highest Shannon index value (Fig. S1).

### Microbial community differences across trophic levels and plant types

**Figure 3.**
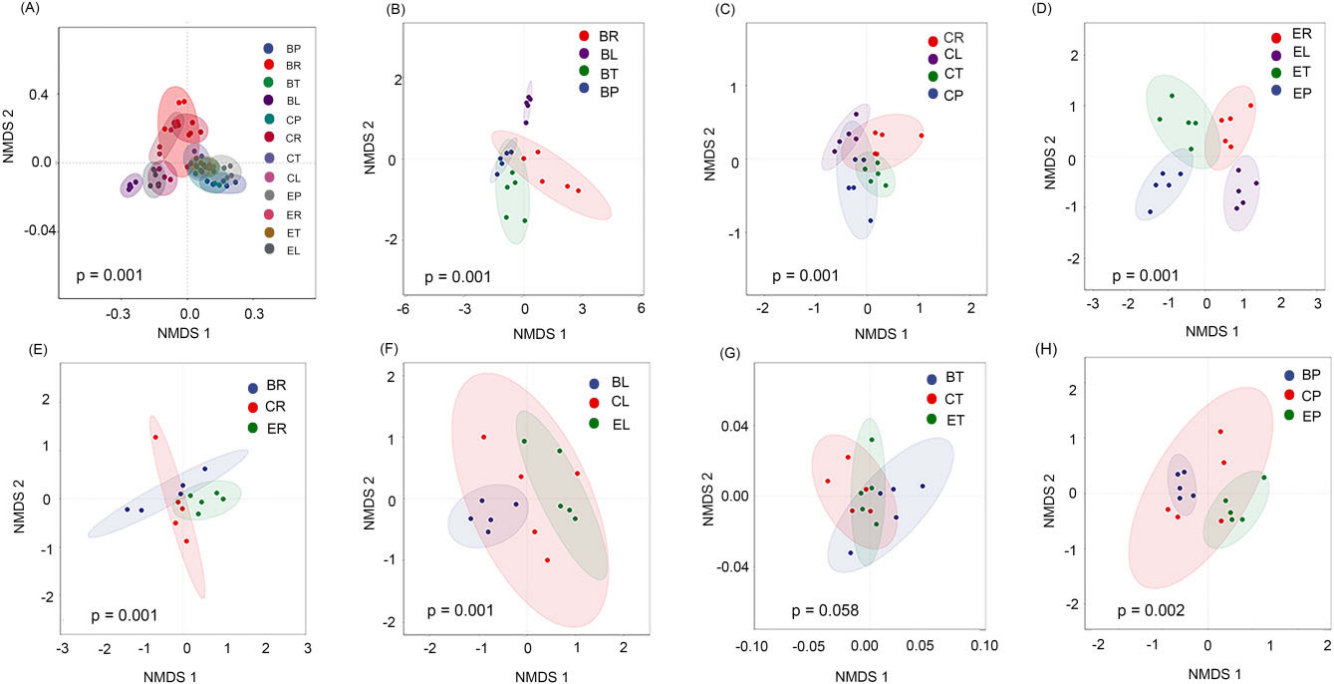
Nonmetric multidimensional scaling ordinations based on weighted Bray–Curtis distance matrices depicting the distribution patterns of bacterial communities along the plant-spider mites–predatory mite continuum. (A) Different trophic levels in diverse plants; different trophic levels in (B) bean, (C) cucumber, and (D) eggplant. (E) Root, (F) leaf, (G) *T. urticae*, and (H) *P. persimilis* on different plants. BR: bean root; CR: cucumber root; ER: eggplant root; BL: bean leaves; CL: cucumber leaves; EL: eggplant leaves; BT: *T. urticae* on bean, CT: *T. urticae* on cucumber; ET: *T. urticae* on eggplant; BP: *P. persimilis* on bean; CP: *P. persimilis* on cucumber; and EP: *P. persimilis* on eggplant.

Each plant harbored a distinct microbial community at each trophic level (bean: *R* = 0.856, *P* = .001; cucumber: *R* = 0.612, *P* = .001; eggplant: *R* = 0.610, *P* = .001, Fig. 3A–D). The root microbiomes of beans and cucumbers were similar (*R* = 0.128, *P* = .142), whereas the root microbiome of eggplants differed significantly from both beans and cucumbers (beans: *R* = 0.372, *P* = .010; cucumbers: *R* = 0.394, *P* = .012, Fig. 3E). Distinct variations were observed in the leaf microbiomes across the three plants (*R* = 0.580, *P* = .010, Fig. 3F), while microbiomes of spider mite on the second trophic level showed no significant differences over the three systems (*R* = 0.694, *P* = .623, Fig. 3G). Notably, the predatory mite microbiomes on cucumbers and beans were not significantly different (*R* = 0.120, *P* = .058), but the microbiome of predatory mite on eggplants was notably distinct (beans versus eggplant: *R* = 0.828, *P* = .007; cucumber versus eggplant: *R* = 0.302, *P* = .021, Fig. 3H).

### Microbial source patterns and network complexity across plant types

**Figure 4.**
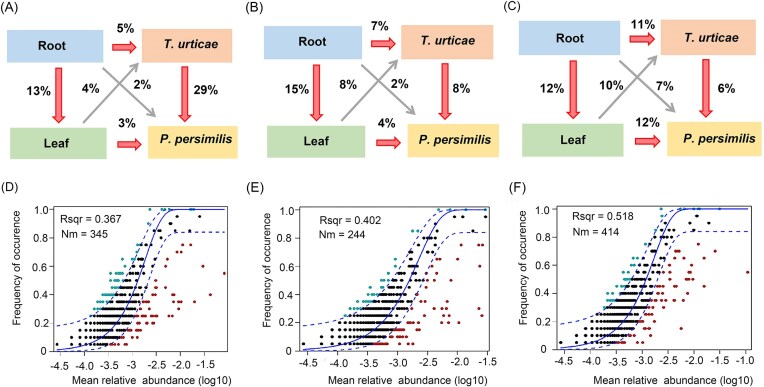
Source model and fit of the NCM of community assembly. Potential sources of bacterial communities of different trophic levels on (A) bean, (B) cucumber, and (C) eggplant. The predicted occurrence frequencies for root–leaf-*T. urticae–P. persimilis* in (D) bean, (E) cucumber, and (F) eggplant system. Dashed lines show 95% confidence intervals. ZOTUs are categorized by their occurrence frequency relative to NCM predictions (higher or lower frequency than expected) as in Sloan et al. (2006). Rsqr indicates the fit to this mode; Nm indicates the migration–dispersal quantity, calculated as the product of metacommunity size (N) and migration rate (m), used to estimate species dispersal capacity between communities.

Based on the SourceTracker model, the microbial community source patterns were consistent across the three systems. Approximately 4%–10% of the leaf bacteria were transferred to the second trophic level (spider mites), while 6%–29% of the bacteria within spider mites were subsequently passed onto predatory mites on the third trophic level (Fig. 4A–C). The core bacteria at each trophic level displayed consistent modular structures across the three tri-trophic systems (bean: 0.87; cucumber: 0.64; and eggplant: 0.80; Fig. S2). The number of “hub nodes” (nodes with high degree values >60 and closeness centrality >0.3) and the average degree were highest in the bean system and lowest in the cucumber system, with a higher average degree and more hub nodes indicating greater network complexity (Table 1).

**Table 1.** The characteristics of bacterial cooccurrence networks on different plants.

Species	Node	Average degree	Diameter	Density	Modularity	Hub node
Bean	248	17.96	9	0.07	0.87	57
Cucumber	249	9.71	11	0.04	0.64	4
Eggplant	250	14.18	9	0.06	0.80	35

### The assembly processes of microbial communities at each trophic level

We applied the Sloan neutral model to investigate microbial community assembly processes across trophic levels in the three plant systems. The NCM effectively estimated the relationship between ZOTU occurrence frequency and relative abundance variations (Fig. 4D–F), explaining 36.7%, 40.2%, and 51.8% of the community variance in the bean, cucumber, and eggplant systems, respectively. Deterministic processes contributed more significantly to community assembly from the roots to predatory mites (*R*² = −0.363–0.298; Fig. S3). The Nm-values were higher in the root microbial communities (Nm = 663 and 975) compared to the leaf communities (Nm = 148 and 639) in the cucumber and eggplant systems. Given a sequence count of 9270 for both samples, the migration rate (m) was estimated at 0.071 and 0.105 in the root, and 0.015 and 0.069 in the leaf, respectively. These findings suggested that microbiota dispersal was more pronounced in the root than in the leaf (Fig. S3).

### Shared ZOTUs distribution and abundance across trophic levels

**Figure 5.**
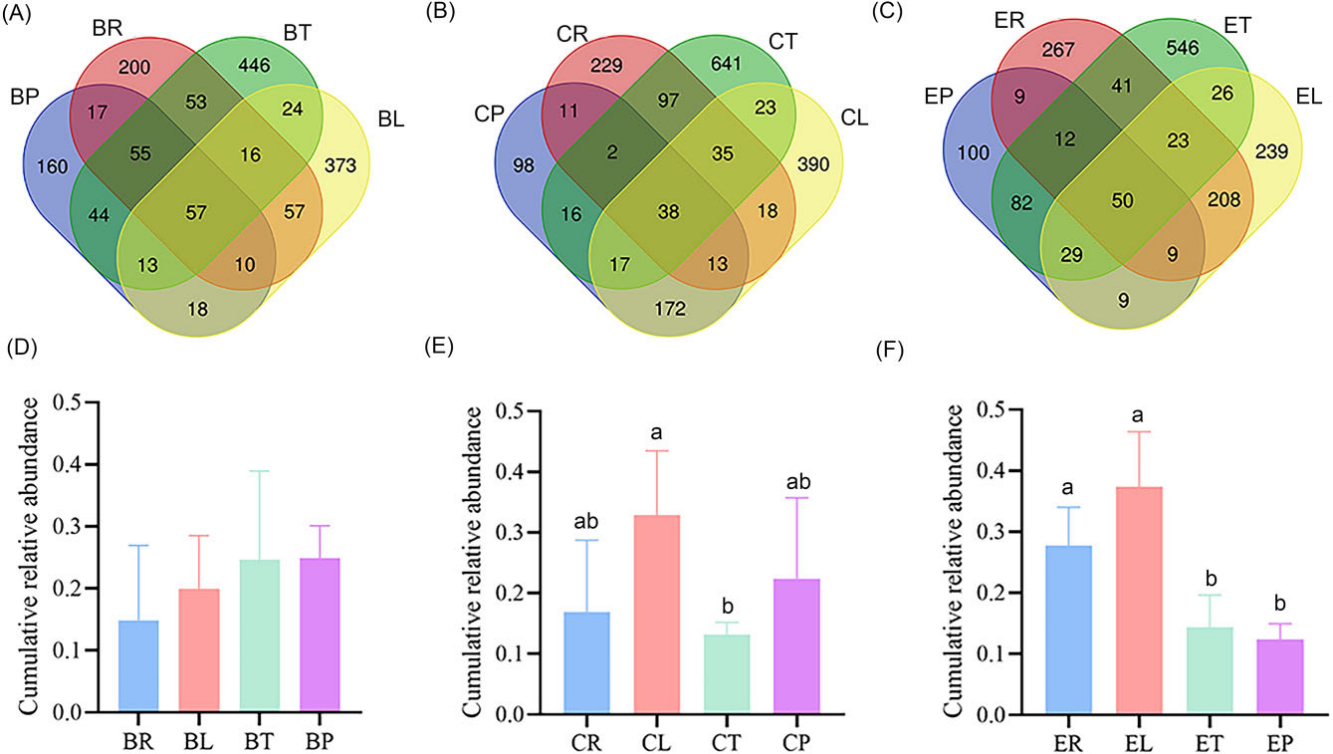
Venn diagrams (A–C) and cumulative relative abundance of shared ZOTUs at each trophic levels (D–F) among different plant. Different lower-case letters indicated significant differences among different trophic levels (*P* < .05).

The microbial composition at different trophic levels of each plant is presented in Fig S4. Bacillales, Micrococcales, and Burkholderiales dominated the microbial communities at all trophic levels. Notably, Rhizobiales were observed in the endosphere of bean plants, and Micrococcales were relatively abundant in the predatory mites on eggplant (Table S5). In the systems of bean, cucumber, and eggplant, there were a total of 57, 38, and 50 shared ZOTUs across each trophic level (with relative abundance > 0.001), respectively (Fig. 5A–C). The majority of these belonged to *Pseudomonas, Bacillus*, and *Staphylococcus* (Tables S1–S3). Notably, the cumulative abundance of shared ZOTUs was highest in leaf tissues in the cucumber and eggplant systems (cucumber: *F*_(3,16)_ = 3.382, *P* = .044; eggplant: *F*_(3,16)_ = 17.958, *P* = .001, Fig. 5D–F).

### Biomarker taxa in mites from different systems and its relationship with fitness

**Figure 6.**
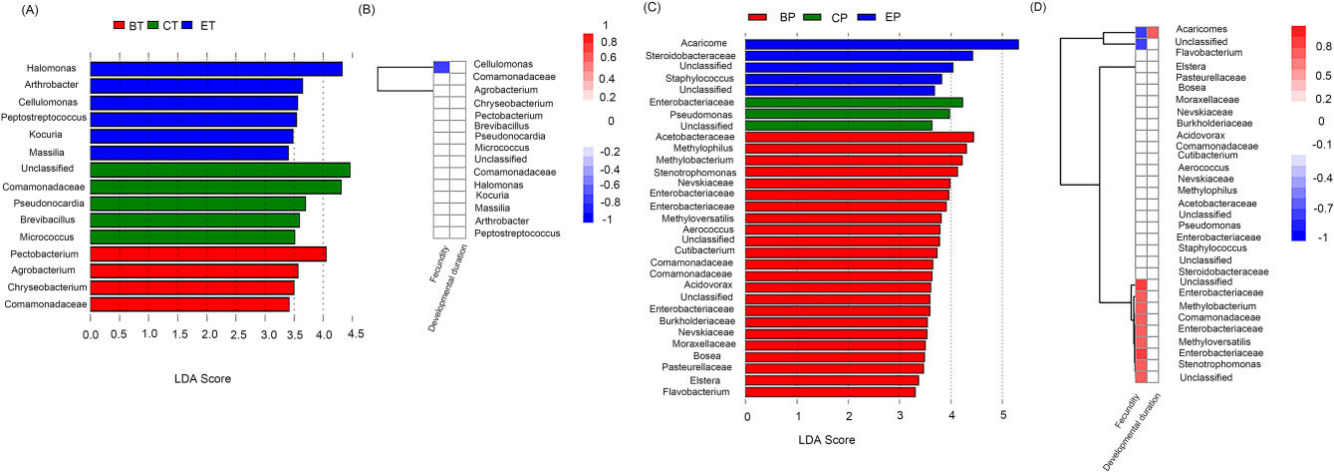
LEfSe analysis and heatmap display the differential bacteria associated with fitness in *T. urticae* (A and B) and *P. persimilis* (C and D). The bar chart from the LEfSe analysis presents the linear discriminant analysis (LDA) scores for biomarkers between mites on different plants, with red indicating spider mites or predatory mites on beans, green for mites on cucumbers, and blue for mites on eggplants. A significance threshold of LDA score 4.0 for spider mites and 5.0 for predatory mites was set, with features exceeding these scores considered significantly different. The heatmap analysis utilized biomarkers identified by LEfSe along with the fitness performance of spider mites and predatory mites on each plant. Blue indicates a negative correlation, while red signifies a positive correlation. The intensity of color reflects the strength of the correlation, ranging from low to high. In (B and D), the solid line on the left illustrates the clustering relationships among different microorganisms. This dendrogram is constructed by grouping similar microorganisms together based on their abundance. The branches of the dendrogram represent hierarchical clustering, with lower-level branches indicating higher similarity between the variables.

Fifteen ZOTUs exhibited significant differences in spider mites across the three different host plants. Specifically, the levels of *Halomonas, Arthrobacter, Cellulomonas, Peptostreptococcus, Kocuria*, and *Massilia* were significantly elevated in spider mites feeding on eggplant, while Comamonadaceae, *Pseudonocardia, Brevibacillus*, and *Micrococcus* were more abundant in those on cucumber. In spider mites from bean system, the contents of *Pectobacterium, Agrobacterium, Chryseobacterium*, and Comamonadaceae were detected at higher levels (Fig. 6A). Through correlation analysis a significant negative correlation was observed exclusively between the genus *Cellulomonas* and spider mite fecundity (Fig. 6B). In this instance for predatory mites, the relative abundance of Micrococcaceae and Burkholderiaceae were significantly higher in samples from eggplants compared to those from beans (Table S4). In total, 31 ZOTUs showed significant variations across different host plants (Fig. 6C). Among these, *Acaricomes* was positively correlated with developmental duration but negatively associated with fecundity. Conversely, *Methylobacterium* and *Stenotrophomonas* were positively correlated with fecundity (Fig. 6D).

**Figure 7.**
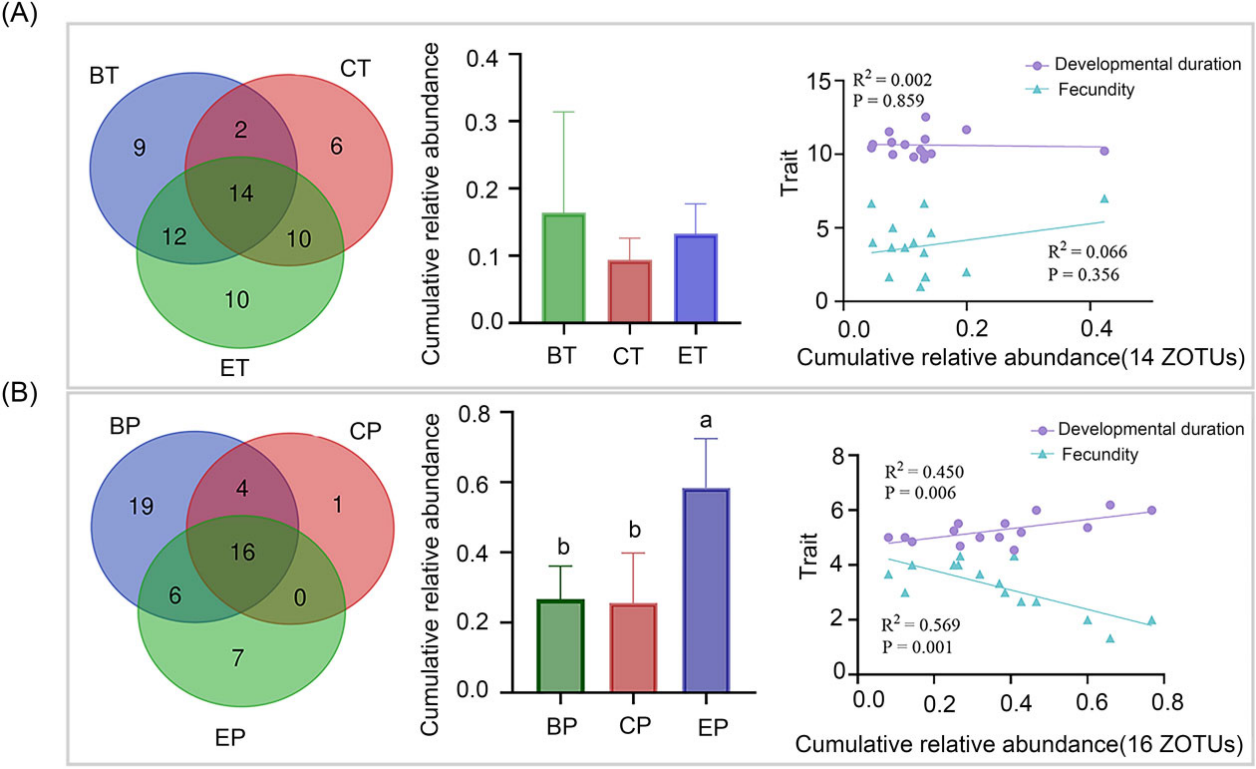
Correlation analyses were conducted between the cumulative relative abundance of shared core ZOTUs and various traits for (A) spider mites and (B) predatory mites on different plants. Venn diagrams (left) illustrate the shared and specific bacterial ZOTUs among mites on different plants, with the central overlapping region representing the shared core ZOTUs of mites across different plants. Bar graphs (middle) display the cumulative relative abundance of these shared core ZOTUs among mites on different plants. Different lower-case letters indicate significant differences within each group (*P* < .05). Correlation (right) between cumulative relative abundance of shared core ZOTU and fitness of mites. The *R*² value indicates the goodness of fit for the model.

Additionally, spider mites across different host plants shared 14 ZOTUs, and the cumulative relative abundance of these ZOTUs did not differ significantly among spider mites from the three systems (*F*_(2,12)_ = 1.350, *P* = .296, Fig. 7A). No notable correlations were observed between the shared ZOTUs and spider mite fitness (Developmental duration: *R*^2^ = 0.020, *P* = .859; Fecundity: *R*^2^ = 0.066, *P* = .356, Fig. 7A). Predatory mites across different systems shared 16 ZOTUs, with the highest cumulative content observed in those from eggplants (*F*_(2,12)_ = 10.689, *P* = .002, Fig. 7B). Notably, this content was significantly positively correlated with developmental duration (*R*^2^ = 0.450, *P* = .006) and negatively correlated with egg production (*R*^2^ = 0.569, *P* = .001, Fig. 7B). Four core ZOTUs were detected in predatory mites colonizing cucumber and bean plants but were absent in those associated with eggplant. These comprised microbial taxa assigned to *Stenotrophomonas, Actinomyces*, and two distinct members of the Enterobacteriaceae family (Fig. 7).

## Discussion

We hypothesized that microbes could flow across trophic levels and that the third trophic level (predatory mites) in a plant–herbivore–carnivore system might be significantly shaped by the first two levels. However, comparative analyses of microbial composition, source tracking, and network modularity revealed many microbial communities at each trophic level were not inherited from the preceding level. This contradicts our previous findings in a prey–predatory mite system, where predatory mite microbiomes were primarily derived from their prey (Yan et al. 2024). This discrepancy may arise from differences in experimental design. The initial study of Yan et al. (2024) used a more controlled laboratory environment with restricted microbial sources (limited to prey–predatory mite interactions), where the prey serves as the main microbial input for predatory mites. In contrast, under greenhouse environments assessed in this study, there is a greater diversity of microbial pool (e.g. soil, air, and phyllosphere microbes), increasing the opportunity for spider mites and predatory mites to acquire microbes through different pathways (e.g. direct plant contact and environmental exposure), thereby reducing the relative contribution of prey-derived microbial transfer. This finding is further supported by experiments conducted by Merlin et al. (2023) in comparable mite systems, where the authors surmised that high functional redundancy within their microbial communities may reduce the need for microbial flow across trophic levels.

The phenomenon of restricted cross-trophic-level microbial transfer observed within the plant–spider mite–predatory mite system is not universally applicable across ecosystems. Research has confirmed the existence of distinct patterns of microbial transfer along trophic levels in typical tri-trophic level systems, such as plant–caterpillar–blue tit and marine food webs (Dion-Phénix et al. 2021, Varg et al. 2022). For instance, studies in marine ecology have demonstrated that microbial communities at lower trophic levels can influence those at higher trophic levels (Varg et al. 2022). Building on findings of Merlin et al. (2023), we propose that restricted cross-trophic microbial transfer constitutes a distinct phenomenon in spider mite–predatory mite systems.

In our study, microbial community assembly in spider mites and predatory mites was predominantly governed by deterministic processes (*R*² < 0.5), consistent with findings in fruit fly larvae, where host filtering, dispersal limitation, and ecological drift shape larval microbiomes (Hendrycks et al. 2025). However, differences in dispersal capacity between systems reveal distinct pathways of deterministic assembly. In fruit fly larvae, extremely low microbial migration rates (m = 0.0002–0.0007) reflect severe dispersal limitations within enclosed fruit microhabitats. Restricted microbial acquisition pathways (vertical transmission and fruit tissue microbiota) necessitate stringent host filtering to preserve functional homeostasis (Singh et al. 2018, Abdelfattah et al. 2021). Conversely, predatory mites exhibited high environmental microbial influx (m = 0.094–0.12), demonstrating continuous environmental inoculation that modifies assembly dynamics. In addition, the combination of high dispersal capacity (elevated Nm) and low model fit (*R*^2^ < 0.5) of the predatory mite microbiome implies that intense deterministic processes override stochastic migration effects in high-dispersal regimes. Specifically, despite frequent microbial immigration (high Nm), deterministic forces-such as local environmental filtering exert dominant control over community structure, leading to systematic deviations from neutral model predictions. This seemingly paradoxical pattern highlights the interplay between niche differentiation (e.g. metabolic specialization) and environmental stressors.

Microorganisms may play a pivotal role in explaining the dietary diversification, characteristics, and adaptability of insects (Hammer and Bowers 2015). For example, short-term adaptation of whiteflies to less suitable hosts correlate with gut microbiome shifts (Santos-Garcia et al. 2020). In this study, the presence of *Methylobacterium* and *Stenotrophomonas*-prominent phyllosphere taxa previously characterized by Legein et al. (2022), was positively correlated with predatory mite fecundity across plant species. *Stenotrophomonas*, a core component of phytoseiid mite microbiomes (Mesostigmata: Phytoseiidae), can be vertically transmitted without adverse effects on mite survival, oviposition, or voracity (Sumner-Kalkun et al. 2023, Yan et al. 2024). Its metabolic versatility likely aids host adaptation to dietary niches (Ozdal et al. 2016). Similarly, *Methylobacterium* may support host energy homeostasis through organic carbon utilization and amino acid conversion (Hu et al. 2013). Conversely, *Cellulomonas*—a genus renowned for cellulolytic activity—was negatively correlated with spider mite fecundity, reflecting potential trade-offs between microbial function and host adaptation (Sun et al. 2018). We also noted the presence of *Acaricome* on eggplants correlated with reduced fecundity of the predatory mite. This may in part reflect that members of this genus, such as *A. phytoseiuli* are pathogenic to *P. persimilis*.

While spider mites across host plants shared 14 core ZOTUs (Fig. 7A), their cumulative abundance showed no significant association with fitness, suggesting these microbes constitute a “basal functional module” maintained by functional redundancy (Merlin et al. 2023). In contrast, the 16 ZOTUs shared by predatory mites were significantly enriched in the eggplant system. This may relate to later outlined differences in eggplant physiochemical properties and or reflect the dietary differences of herbivorous versus carnivorous mites, where differences in intestinal pH and proteases will alter the availability of nutrients to their associated microflora (Lazarević and Janković‐Tomanić 2015, Santamaría et al. 2015). Collectively, microbial functional divergence reflects both host adaptation to plant-specific chemical environments and potential drivers of dietary specialization through metabolic plasticity.

As the primary trophic level, plants play a pivotal regulatory role in shaping higher trophic levels. Most studies on plant species diversity effects have focused on static, unidirectional (bottom-up) frameworks (Abdala-Roberts et al. 2019). We observed significant shifts in predatory mite microbiomes on eggplants, seemingly unaffected by direct microbial inputs from lower trophic levels. Given limited microbial transfer, we emphasize the greater influence of plant intrinsic traits (e.g. nutrients and secondary metabolites) on secondary and tertiary trophic levels. In bamboo-feeding insects, microbial abundance correlates with nutrient concentrations (Huang et al. 2021). Cucurbit-specialized insects harbor shared Enterobacteriaceae taxa linked to cucurbitacin degradation (Zhong et al. 2022). Similarly, high metabolite content in Solanaceae plants (e.g. eggplants) likely drives microbiome composition, with cascading effects across trophic levels (Hanifah et al. 2018).

Based on the results of this study, we propose a “plant traits-host filtering-microbial function” framework, advancing our understanding of multitrophic interactions in open ecosystems. We speculate microbial cross-trophic transfer efficiency is synergistically regulated by host ecological niches and plant chemical traits (e.g. secondary metabolites and nutrient stoichiometry), especially in the eggplant system. Meanwhile, the universality of deterministic processes arises from hosts actively converting stochastic environmental inputs into functionally structured communities through physiological and ecological filtering. This framework can be used to inform precision biocontrol strategies: targeted enhancement of vertically transmitted symbionts (e.g. *Stenotrophomonas* with plant metabolite-degrading capabilities) or optimization of host plant chemical profiles (e.g. Solanaceae alkaloid modulation) that in turn could amplify predatory mite adaptability and pest suppression efficacy. This scenario would bridge the gap between ecological theory with agricultural innovation.

## Supplementary Material

fiaf065_Supplemental_File

## References

[bib1] Abdala-Roberts L, Puentes A, Finke DL et al. Tri-trophic interactions: bridging species, communities and ecosystems. Ecol Lett. 2019;22:2151–67. 10.1111/ele.13392. 31631502 PMC6899832

[bib2] Abdelfattah A, Freilich S, Bartuv R et al. Global analysis of the apple fruit microbiome: are all apples the same?. Environ Microbiol. 2021;23:6038–55. 10.1111/1462-2920.15469. 33734550 PMC8596679

[bib3] Bastian M, Heymann S, Jacomy M. Gephi: an open source software for exploring and manipulating networks. In: Proceedings of the Third International Conference on Weblogs and Social Media. San Jose, CA: AAAI Publications, 2009.

[bib4] Berasategui A, Breitenbach N, García-Lozano M et al. The leaf beetle *Chelymorpha alternans* propagates a plant pathogen in exchange for pupal protection. Curr Biol. 2022;32:4114–4127.e6. 10.1016/j.cub.2022.07.065. 35987210

[bib5] Bolger AM, Lohse M, Usadel B. Trimmomatic: a flexible trimmer for Illumina sequence data. Bioinformatics. 2014;30:2114–20. 10.1093/bioinformatics/btu170. 24695404 PMC4103590

[bib6] Brady CM, Asplen MK, Desneux N et al. Worldwide populations of the aphid *Aphis craccivora* are infected with diverse facultative bacterial symbionts. Microb Ecol. 2014;67:195–204. 10.1007/s00248-013-0314-0. 24233285

[bib7] Chrostek E, Pelz-Stelinski K, Hurst GDD et al. Horizontal transmission of intracellular insect symbionts via plants. Front Microbiol. 2017;8:2237. 10.3389/fmicb.2017.02237. 29234308 PMC5712413

[bib8] Cole JR, Wang Q, Fish JA et al. Ribosomal Database Project: data and tools for high throughput rRNA analysis. Nucl Acids Res. 2014;42:D633–42. 10.1093/nar/gkt1244. 24288368 PMC3965039

[bib9] Dion-Phénix H, Charmantier A, de Franceschi C et al. Bacterial microbiota similarity between predators and prey in a blue tit trophic network. ISME J. 2021;15:1098–107. 10.1038/s41396-020-00836-3. 33580209 PMC8115664

[bib10] Edgar RC, Flyvbjerg H. Error filtering, pair assembly and error correction for next-generation sequencing reads. Bioinformatics. 2015;31:3476–82. 10.1093/bioinformatics/btv401. 26139637

[bib11] Edgar RC. UPARSE: highly accurate OTU sequences from microbial amplicon reads. Nat Methods. 2013;10:996–8. 10.1038/nmeth.2604. 23955772

[bib12] Frago E, Dicke M, Godfray HC. Insect symbionts as hidden players in insect-plant interactions. Trends Ecol Evol. 2012;27:705–11. 10.1016/j.tree.2012.08.013. 22985943

[bib13] Frago E, Zytynska S. Impact of herbivore symbionts on parasitoid foraging behaviour. Curr Opin Insect Sci. 2023;57:101027. 10.1016/j.cois.2023.101027. 36990151

[bib14] Gomes SIF, Kielak AM, Hannula SE et al. Microbiomes of a specialist caterpillar are consistent across different habitats but also resemble the local soil microbial communities. Anim Microbiome. 2020;2:37. 10.1186/s42523-020-00055-3. 33499994 PMC7807420

[bib15] Grbić M, Van Leeuwen T, Clark RM et al. The genome of *Tetranychus urticae* reveals herbivorous pest adaptations. Nature. 2011;479:487–92. 10.1038/nature10640. 22113690 PMC4856440

[bib16] Gu X, Ross PA, Gill A et al. A rapidly spreading deleterious aphid endosymbiont that uses horizontal as well as vertical transmission. Proc Natl Acad Sci USA. 2023;120:e2217278120. 10.1073/pnas.2217278120. 37094148 PMC10161079

[bib17] Hammer TJ, Bowers MD. Gut microbes may facilitate insect herbivory of chemically defended plants. Oecologia. 2015;179:1–14. 10.1007/s00442-015-3327-1. 25936531

[bib18] Hanifah A, Maharijaya A, Putri SP et al. Untargeted metabolomics analysis of eggplant (*Solanum melongena* L.) fruit and its correlation to fruit morphologies. Metabolites. 2018;8:49. 10.3390/metabo8030049. 30200482 PMC6160926

[bib19] Hannula SE, Zhu F, Heinen R et al. Foliar-feeding insects acquire microbiomes from the soil rather than the host plant. Nat Commun. 2019;10:1254. 10.1038/s41467-019-09284-w. 30890706 PMC6425034

[bib20] Hendrycks W, Mullens N, Bakengesa J et al. Deterministic and stochastic effects drive the gut microbial diversity in cucurbit-feeding fruit flies (Diptera, Tephritidae). PLoS One. 2025;20:e0313447. 10.1371/journal.pone.0313447. 39854335 PMC11759365

[bib21] Hu X, Wang C, Chen H et al. Differences in the structure of the gut bacteria communities in development stages of the Chinese white pine beetle (*Dendroctonus armandi*). Int J Mol Sci. 2013;14:21006–20. 10.3390/ijms141021006. 24145750 PMC3821655

[bib22] Huang K, Wang J, Huang J et al. Host phylogeny and diet shape gut microbial communities within bamboo-feeding insects. Front Microbiol. 2021;12:633075. 10.3389/fmicb.2021.633075. 34239504 PMC8260032

[bib23] Kikuchi Y, Hayatsu M, Hosokawa T et al. Symbiont-mediated insecticide resistance. Proc Natl Acad Sci USA. 2012;109:8618–22. 10.1073/pnas.1200231109. 22529384 PMC3365206

[bib24] Kim DR, Cho G, Jeon CW et al. A mutualistic interaction between *Streptomyces* bacteria, strawberry plants and pollinating bees. Nat Commun. 2019;10:4802. 10.1038/s41467-019-12785-3. 31641114 PMC6805876

[bib25] Knight R, Kelley ST. Bayesian community-wide culture-independent microbial source tracking. Nat Methods. 2011;8:761–3. 10.1038/nmeth.1650. 21765408 PMC3791591

[bib26] Lazarević J, Janković-Tomanić M. Dietary and phylogenetic correlates of digestive trypsin activity in insect pests. Entomol Exp Appl. 2015;157:123–51. 10.1111/eea.12349.

[bib27] Legein M, Smets W, Wuyts K et al. The greenhouse phyllosphere microbiome and associations with introduced bumblebees and predatory mites. Microbiol Spectr. 2022;10:e0175522. 10.1128/spectrum.01755-22. 35862945 PMC9431046

[bib28] Li YH, Ahmed MZ, Li SJ et al. Plant-mediated horizontal transmission of *Rickettsia* endosymbiont between different whitefly species. FEMS Microbiol Ecol. 2017;93:fix138. 10.1093/femsec/fix138. 29069333

[bib29] Li Z, Zhao W, Jiang Y et al. New insights into biologic interpretation of bioinformatic pipelines for fish eDNA metabarcoding: a case study in Pearl River estuary. J Environ Manage. 2024;368:122136. 10.1016/j.jenvman.2024.122136. 39128344

[bib30] Merlin BL, Moraes GJ, Cônsoli FL. The microbiota of a mite prey-predator system on different host plants are characterized by dysbiosis and potential functional redundancy. Microb Ecol. 2023;85:1590–607. 10.1007/s00248-022-02032-6. 35543735

[bib31] Molinari S, Leonetti P. Resistance to plant parasites in tomato is induced by soil enrichment with specific bacterial and fungal *Rhizosphere* microbiome. Int J Mol Sci. 2023;24:15416. 10.3390/ijms242015416. 37895095 PMC10607013

[bib32] Najafabadi SS, Shoushtari RV, Zamani AA et al. Life parameters of *Tetranychus urticae* (Acari: tetranychidae) on six common bean cultivars. J Econ Entomol. 2014;107:614–22. 10.1603/EC11205. 24772541

[bib33] Ozdal M, Ozdal OG, Alguri OF. Isolation and characterization of α-endosulfan degrading bacteria from the microflora of cockroaches. Pol J Microbiol. 2016;65:63–8. 10.5604/17331331.1197325. 27281995

[bib34] Pekas A, Palevsky E, Sumner JC et al. Comparison of bacterial microbiota of the predatory mite *Neoseiulus cucumeris* (Acari: phytoseiidae) and its factitious prey *Tyrophagus putrescentiae* (Acari: acaridae). Sci Rep. 2017;7:2. 10.1038/s41598-017-00046-6. 28127053 PMC5428342

[bib35] Rasmann S, Bennett A, Biere A et al. Root symbionts: powerful drivers of plant above-and below ground indirect defenses. Insect Sci. 2017;24:947–60. 10.1111/1744-7917.12464. 28374534

[bib36] Santamaría ME, González-Cabrera J, Martínez M et al. Digestive proteases in bodies and faeces of the two-spotted spider mite, *Tetranychus urticae*. J Insect Physiol. 2015;78:69–77. 10.1016/j.jinsphys.2015.05.002. 25960286

[bib37] Santos-Garcia D, Mestre-Rincon N, Zchori-Fein E et al. Inside out: microbiota dynamics during host-plant adaptation of whiteflies. ISME J. 2020;14:847–56. 10.1038/s41396-019-0576-8. 31896788 PMC7031279

[bib38] Schädler M, Ballhorn DJ. Beneficial soil microbiota as mediators of the plant defensive phenotype and aboveground plant-herbivore interactions. Prog Biomater. 2016;78:305–43.

[bib39] Segata N, Izard J, Waldron L et al. Metagenomic biomarker discovery and explanation. Genome Biol. 2011;12:R60. 10.1186/gb-2011-12-6-r60. 21702898 PMC3218848

[bib40] Singh P, Santoni S, This P et al. Genotype-environment interaction shapes the microbial assemblage in grapevine’s phyllosphere and carposphere: an NGS approach. Microorganisms. 2018;6:96. 10.3390/microorganisms6040096. 30248973 PMC6313654

[bib41] Sloan WT, Lunn M, Woodcock S et al. Quantifying the roles of immigration and chance in shaping prokaryote community structure. Environ Microbiol. 2006;8:732–40. 10.1111/j.1462-2920.2005.00956.x. 16584484

[bib42] Sumner-Kalkun JC, Baxter I, Perotti MA. Bacterial microbiota of three commercially mass-reared predatory mite species (Mesostigmata: phytoseiidae): pathogenic and beneficial interactions. Front Arachn Sci. 2023;2:1242716. 10.3389/frchs.2023.1242716.

[bib43] Sun X, Li J, Du J et al. *Cellulomonas macrotermitis* sp. nov., a chitinolytic and cellulolytic bacterium isolated from the hindgut of a fungus-growing termite. Antonie Van Leeuwenhoek. 2018;111:471–8. 10.1007/s10482-017-0968-6. 29090357

[bib44] Tao LL, Mark DH, de Roode JC. Microbial root mutualists affect the predators and pathogens of herbivores above ground: mechanisms, magnitudes, and missing links. Front Ecol Evol. 2017;5. 10.3389/fevo.2017.00160.

[bib45] Ushio M, Yamasaki E, Takasu H et al. Microbial communities on flower surfaces act as signatures of pollinator visitation. Sci Rep. 2015;5:8695. 10.1038/srep08695. 25733079 PMC4346974

[bib46] Varg JE, Outomuro D, Kunce W et al. Microplastic exposure across trophic levels: effects on the host-microbiota of freshwater organisms. Environ Microbiome. 2022;17:36. 10.1186/s40793-022-00429-x. 35794681 PMC9258161

[bib47] Yan H, Wang ED, Wei GS et al. Both host and diet shape bacterial communities of predatory mites. Insect Sci. 2024;31:551–61. 10.1111/1744-7917.13253. 37469127

[bib48] Yan JY, Zhang B, Li GT et al. Bacterial communities in predatory mites are associated with species and diet types. BioControl. 2021;66:803–11. 10.1007/s10526-021-10112-8.

[bib49] Zhang SK, Li ZK, Shu JP et al. Soil-derived bacteria endow *Camellia weevil* with more ability to resist plant chemical defense. Microbiome. 2022;10:97. 10.1186/s40168-022-01290-3. 35752840 PMC9233397

[bib50] Zhong Y, Xun W, Wang X et al. Root-secreted bitter triterpene modulates the rhizosphere microbiota to improve plant fitness. Nat Plants. 2022;8:887–96. 10.1038/s41477-022-01201-2. 35915145

[bib51] Zhou SX, Lu X, Yu FL et al. Functional response and predation preference of *Phytoseiulus persimilis* to different stages of *Tetrarcychus urticae*. J Northeast Agric Sci. 2024;49:50–4.

[bib52] Zhu YX, Song YL, Hoffmann AA et al. A change in the bacterial community of spider mites decreases fecundity on multiple host plants. Microbiologyopen. 2019;8:e00743. 10.1002/mbo3.743. 30311439 PMC6562136

