# Supplementary material for: Microbial dynamics across tri-trophic systems: insights from plant–herbivore–predator interactions
Source: FEMS Microbiol Ecol. 2025 Jun 18;101(7):fiaf065. doi: 10.1093/femsec/fiaf065 (PMC12199696; doi:10.1093/femsec/fiaf065)
Supplement: fiaf065_Supplemental_File [file fiaf065_supplemental_file.docx]

**Supplementary Information for**

**Microbial dynamics across tritrophic systems: Insights from Plant-mite-predator interactions**

Hong Yan^1,2^, Endong Wang^2,3^, Xuenong Xu^2,3^, Guo-Shu Wei^1^*, Bo Zhang^2,3^*

*1 College of Plant Protection, Hebei Agricultural University, Baoding 071000, China*

*2 State Key Laboratory for Biology of Plant Diseases and Insect Pests, Institute of Plant Protection, Chinese Academy of Agricultural Sciences, Beijing 100193, China*

*3 Key Laboratory of Natural Enemies Insects, Ministry of Agriculture and Rural Affairs, Beijing 100193, China*

**Corresponding authors: zhangbo05@caas.cn (B. Zhang), weiguoshu03@aliyun.com (G.-S. Wei)*

Correspondence: Guo-Shu Wei, College of Plant Protection, Hebei Agricultural University, Baoding 071000, Hebei Province, China. Tel: +86 010 7528148; email: weiguoshu03@aliyun.com; Bo Zhang, Institute of Plant Protection, Chinese Academy of Agricultural Sciences, Beijing 100193, China. Email: zhangbo05@caas.cn.

**The file includes:**

Figure S1-S4

Table S1-S5


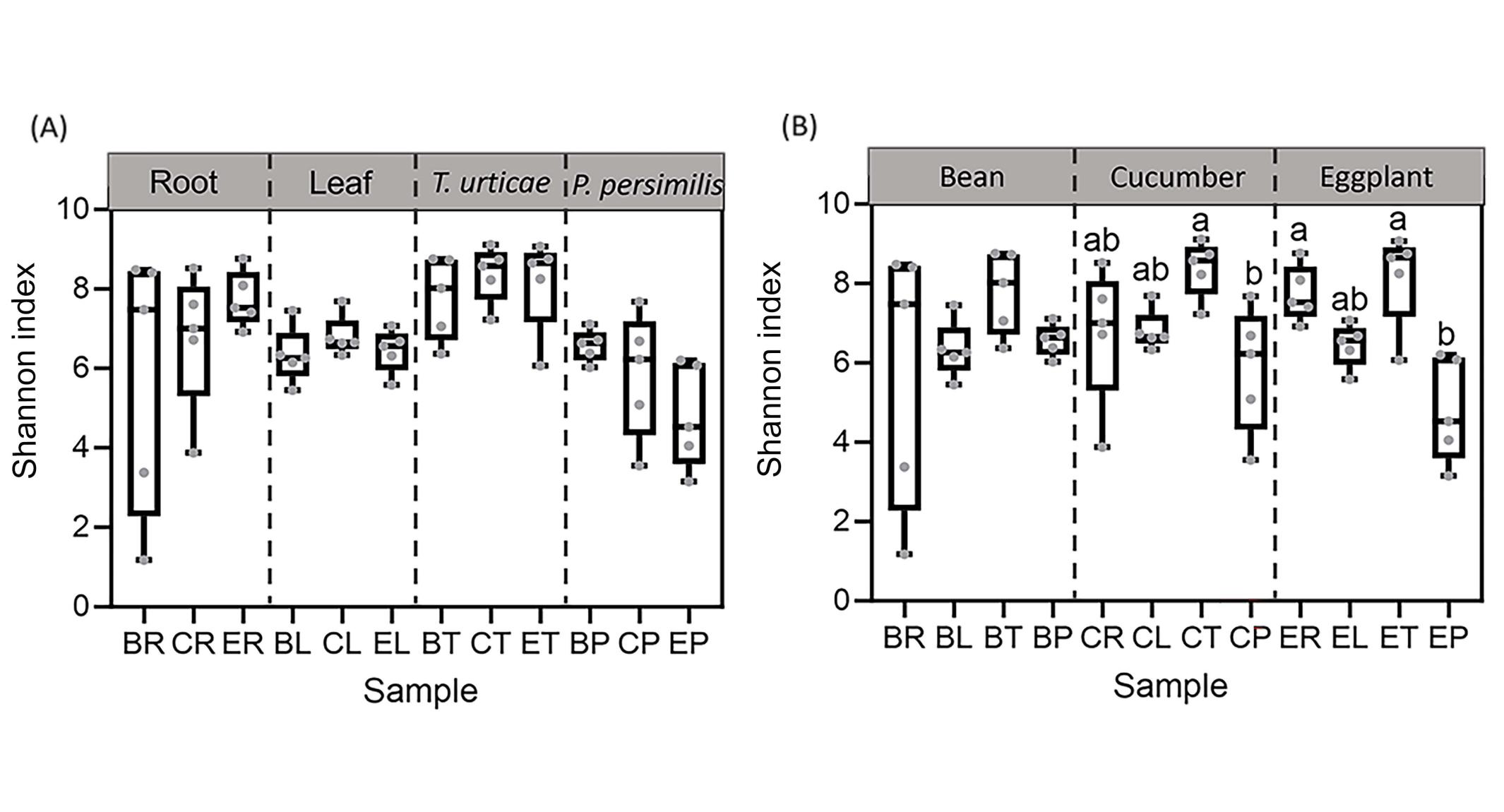


**Fig S1.** Alpha diversity indices of bacterial communities. (A) The same trophic level of different plants; (B) The various trophic levels of same plants. BR: The root of the bean; CR: The root of the cucumber; ER: The root of the eggplant; BL: The leaves of bean; CL: The leaves of cucumber; EL: The leaves of leaves; BT: *T. urticae* on bean, CT: *T. urticae* on cucumber; ET: *T. urticae* on eggplant; BP: *P. persimilis* on bean; CP: *P. persimilis* on cucumber; EP: *P. persimilis* on eggplant. ns, not significant. Different lower-case letters indicated significant differences among various trophic levels (*p* < 0.05).


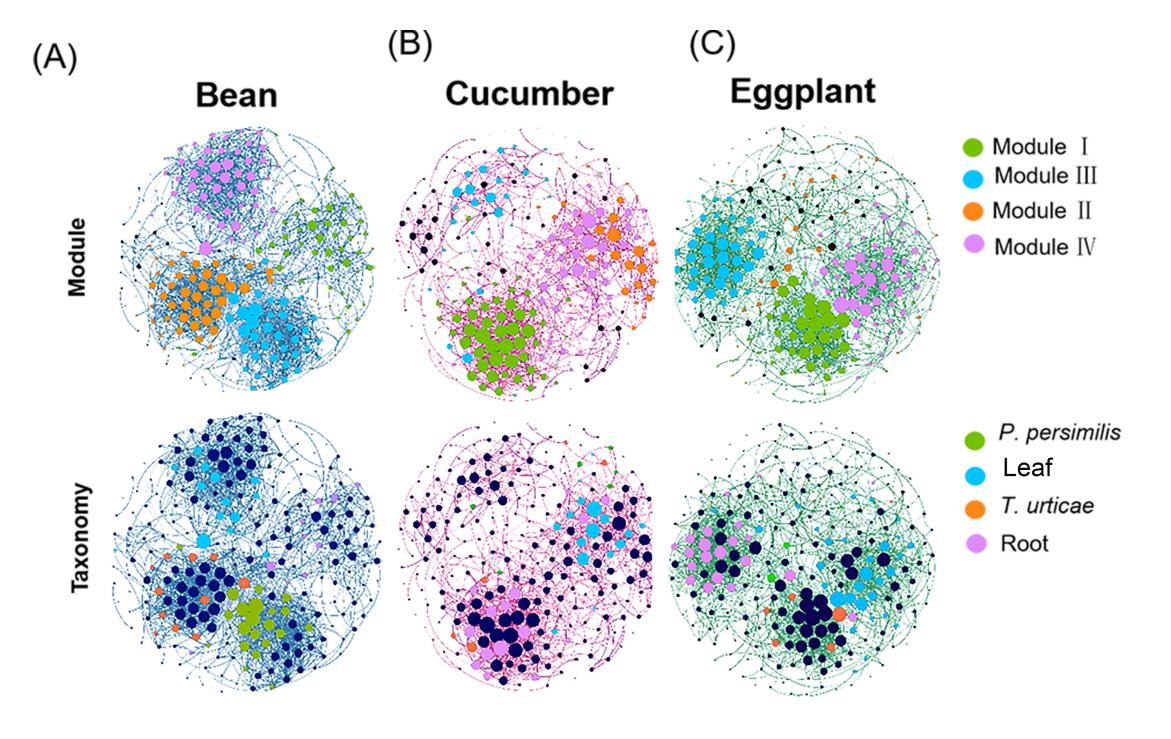


**Fig S2**. Spatial dynamics of bacterial interkingdom networks. The core ZOTUs (definition: ZOTU abundance ≥0.001 per sample; each group had at least 3 replicated abundances greater than 0.001) of different trophic levels on each plant were used to create a network. (A) bean, (B) cucumber, and (C) eggplant system.

**
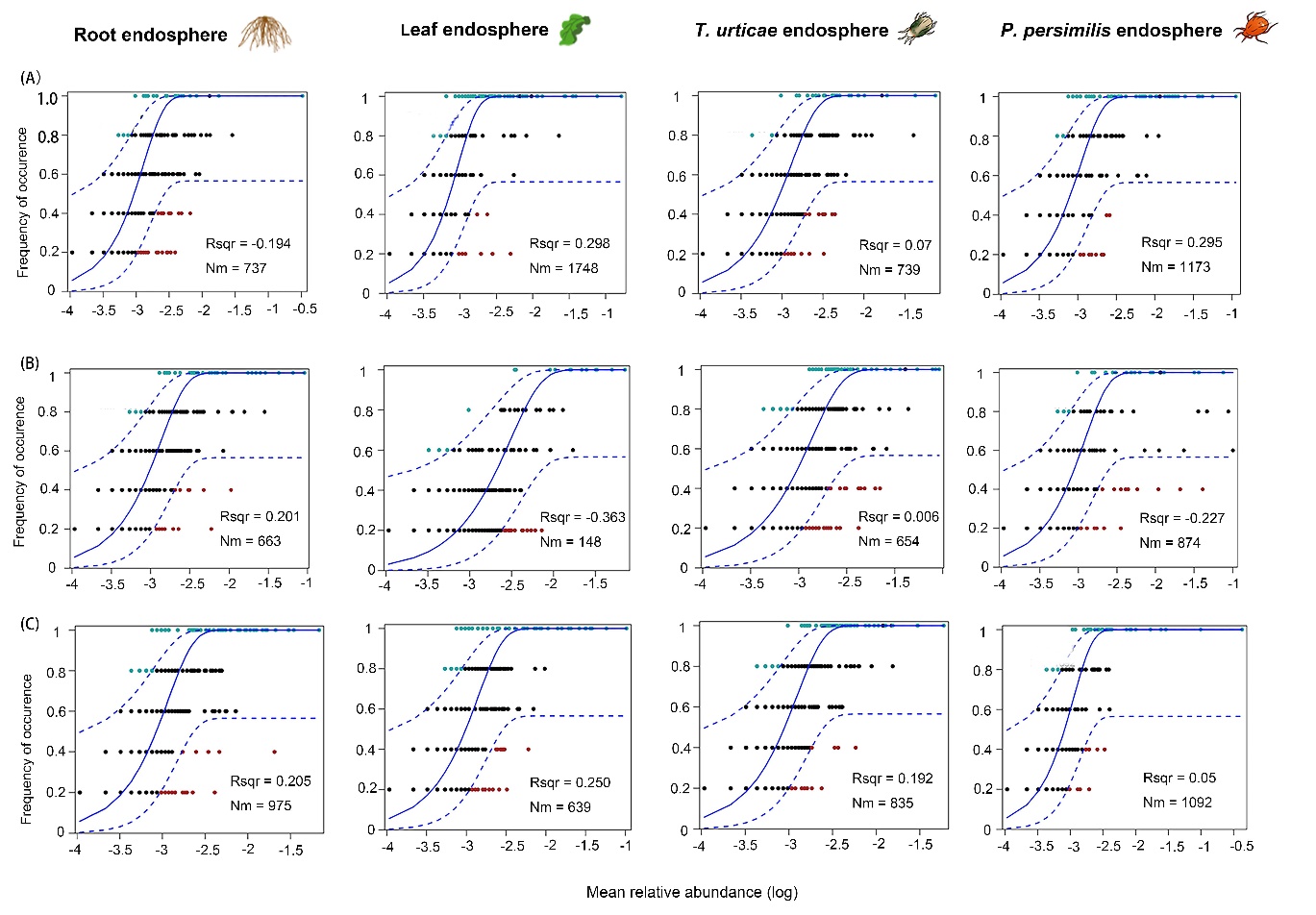
**

**Fig S3.** The neutral community model (NCM) of community assembly across different trophic levels. The predicted occurrence frequencies for (A) bean, (B) cucumber, and (C) eggplant system. The solid blue lines indicate the best fit to the NCM as in Sloan et al., and the dashed blue lines represent 95% confidence intervals around the model prediction. ZOTUs that occur more or less frequently than predicted by the NCM are shown in different colors. Rsqr indicates the fit to this mode; Nm indicates the migration-dispersal quantity, calculated as the product of metacommunity size (N) and migration rate (m), used to estimate species dispersal capacity between communities.

**
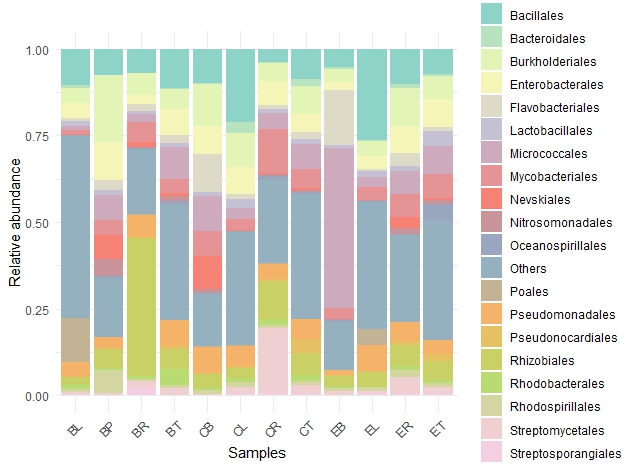
**

**Fig S4.** The relative abundance distribution of microbial communities across different samples. The x-axis denotes individual samples, and the y-axis represents relative abundance. Distinct color codes correspond to bacterial taxa at the order level.

Table S1. The genus of shared ZOTU at each trophic level in the bean system.

| ZOTU | Genus |
| --- | --- |
| ZOTU_11, 74, 69, 167, 513 | *Staphylococcus* |
| ZOTU_164 | *Agrobacterium* |
| ZOTU_231, 67, 559 | *Streptococcus* |
| ZOTU_130, 297 | *Neisseria* |
| ZOTU_26 | *Acinetobacter* |
| ZOTU_119, 79, 62, 248, 14, 71, 18 | *Pseudomonas* |
| ZOTU_40, 88 | *Delftia* |
| ZOTU_86, 16 | Enterobacteriaceae |
| ZOTU_22 | *Brevundimonas* |
| ZOTU_270 | Alcaligenaceae |
| ZOTU_208 | Moraxellaceae |
| ZOTU_76, 80 | *Actinomyces* |
| ZOTU_13, 27 | *Cutibacterium* |
| ZOTU_38 | *Methylophilus* |
| ZOTU_78, 42 | *Stenotrophomonas* |
| ZOTU_9 | *Serratia* |
| ZOTU_95 | Lawsonellaceae |
| ZOTU_63, 100 | *Streptomyces* |
| ZOTU_46 | *Ralstonia* |
| ZOTU_49 | Enterobacterales |
| ZOTU_4810 | Gammaproteobacteria |
| ZOTU_19 | Bacillaceae_1 |
| ZOTU_29, 75 | *Corynebacterium* |
| ZOTU_258 | *Actinobacteria* |
| ZOTU_101 | *Rhodococcus* |
| ZOTU_188 | Erwiniaceae |
| ZOTU_77 | Pseudomonadaceae |
| ZOTU_85 | *Mycobacterium* |
| ZOTU_58 | *Enterococcus* |
| ZOTU_52 | *Alcaligenes* |
| ZOTU_157 | *Sphingomonas* |
| ZOTU_21 | Acetobacteraceae |
| ZOTU_30, 8 | *Bacillus* |
| ZOTU_244 | *Nosocomiicoccus* |
| ZOTU_89 | *Schaalia* |
| ZOTU_126 | Corynebacteriaceae |

Note: Some ZOTUs that were not identified to the genus level are represented by their family names.

Table S2. The genus of shared ZOTU at each trophic level in the cucumber system.

| ZOTU | Genus |
| --- | --- |
| ZOTU_11, 74, 69, 167 | *Staphylococcus* |
| ZOTU_163, 222 | *Paracoccus* |
| ZOTU_130, 297, 174 | *Neisseria* |
| ZOTU_26 | *Acinetobacter* |
| ZOTU_208 | Moraxellaceae |
| ZOTU_76 | *Actinomyces* |
| ZOTU_13, 27 | *Cutibacterium* |
| ZOTU_78, 42 | *Stenotrophomonas* |
| ZOTU_9 | *Serratia* |
| ZOTU_95 | Lawsonellaceae |
| ZOTU_63 | *Streptomyces* |
| ZOTU_46 | *Ralstonia* |
| ZOTU_49 | Enterobacterales |
| ZOTU_19 | Bacillaceae_1 |
| ZOTU_25 | *Methylobacterium* |
| ZOTU_29 | *Corynebacterium* |
| ZOTU_67 | *Streptococcus* |
| ZOTU_62, 18, 71, 14 | *Pseudomonas* |
| ZOTU_77 | Pseudomonadaceae |
| ZOTU_52 | *Alcaligenes* |
| ZOTU_21 | Acetobacteraceae |
| ZOTU_30, 8 | *Bacillus* |
| ZOTU_322 | *Rhizobium* |
| ZOTU_116 | *Micrococcus* |
| ZOTU_89 | *Schaalia* |
| ZOTU_141 | *Geodermatophilus* |

Note: Some ZOTUs that were not identified to the genus level are represented by their family names.

Table S3. The genus of shared ZOTU at each trophic level in the eggplant system.

| ZOTU | Genus |
| --- | --- |
| ZOTU_304 | *Gemella* |
| ZOTU_11, 217, 74, 69, 167 | *Staphylococcus* |
| ZOTU_130 | *Neisseria* |
| ZOTU_119, 18, 71,14,190, 62, | *Pseudomonas* |
| ZOTU_40 | *Delftia* |
| ZOTU_22 | *Brevundimonas* |
| ZOTU_208 | Moraxellaceae |
| ZOTU_76 | *Actinomyces* |
| ZOTU_51 | *Actinoallomurus* |
| ZOTU_115 | *Kocuria* |
| ZOTU_224, 59 | *Microbacterium* |
| ZOTU_4 | *Rhizobium* |
| ZOTU_13, 27 | *Cutibacterium* |
| ZOTU_78, 42 | *Stenotrophomonas* |
| ZOTU_9 | *Serratia* |
| ZOTU_95 | Lawsonellaceae |
| ZOTU_136 | *Acinetobacter* |
| ZOTU_580, 234, 29, 75, 123 | *Corynebacterium* |
| ZOTU_117 | *Brevibacillus* |
| ZOTU_63, 100 | *Streptomyces* |
| ZOTU_49 | Enterobacterales |
| ZOTU_264 | *Aerococcus* |
| ZOTU_19 | Bacillaceae |
| ZOTU_24 | Enterobacteriaceae |
| ZOTU_81 | Corynebacteriaceae |
| ZOTU_67 | *Streptococcus* |
| ZOTU_52 | *Alcaligenes* |
| ZOTU_294 | *Glutamicibacter* |
| ZOTU_21 | Acetobacteraceae |
| ZOTU_30, 8 | *Bacillus* |
| ZOTU_236 | *Pseudarthrobacter* |
| ZOTU_89 | *Schaalia* |

Note: Some ZOTUs that were not identified to the genus level are represented by their family names.

Table S4. Family-level bacterial composition (mean ± SE) across predatory mites on different plants. BP: *P. persimilis* on bean; CP: *P. persimilis* on cucumber; EP: *P. persimilis* on eggplant. Different lower-case letters indicated significant differences among (*p* < 0.05)

| Level | BP | CP | EP |
| --- | --- | --- | --- |
| Micrococcaceae | 0.06±0.05b | 0.09±0.07b | 0.45±0.22a |
| Weeksellaceae | 0.02±0.01 | 0.11±0.23 | 0.15±0.24 |
| Nevskiaceae | 0.07±0.06 | 0.09±0.17 | 0.001±0.001 |
| Pseudomonadaceae | 0.02±0.01 | 0.07±0.11 | 0.01±0.01 |
| Corynebacteriaceae | 0.04±0.01 | 0.07±0.11 | 0.02±0.01 |
| Burkholderiaceae | 0.14±0.05a | 0.09±0.12ab | 0.01±0.004b |
| Enterobacteriaceae | 0.08±0.03 | 0.06±0.10 | 0.01±0.002 |
| Bacillaceae_1 | 0.03±0.01 | 0.07±0.09 | 0.02±0.01 |
| Acetobacteraceae | 0.06±0.08 | 0.01±0.01 | 0.01±0.01 |
| Others | 0.49±0.05 | 0.36±0.18 | 0.31±0.10 |

**Table S5** Order-level bacterial composition (mean ± SE) across distinct trophic levels. BR: The root of the bean; CR: The root of the cucumber; ER: The root of the eggplant; BL: The leaves of bean; CL: The leaves of cucumber; EL: The leaves of leaves; BT: *T. urticae* on bean, CT: *T. urticae* on cucumber; ET: *T. urticae* on eggplant; BP: *P. persimilis* on bean; CP: *P. persimilis* on cucumber; EP: *P. persimilis* on eggplant.

|  | BR | BL | BT | BP | CR | CL | CT | CP | ER | EL | ET | EP |
| --- | --- | --- | --- | --- | --- | --- | --- | --- | --- | --- | --- | --- |
| Rhizobiales | 0.40±0.43 | 0.02±0.01 | 0.06±0.02 | 0.06±0.03 | 0.11±0.06 | 0.03±0.01 | 0.07±0.02 | 0.04±0.06 | 0.07±0.04 | 0.04±0.04 | 0.05±0.02 | 0.03±0.02 |
| Streptomycetales | 0.02±0.01 | 0.01±0.00 | 0.02±0.01 | 0.01±0.01 | 0.18±0.35 | 0.02±0.02 | 0.03±0.03 | 0.00±0.00 | 0.02±0.01 | 0.01±0.01 | 0.02±0.01 | 0.01±0.02 |
| Micrococcales | 0.03±0.01 | 0.01±0.00 | 0.09±0.03 | 0.07±0.05 | 0.05±0.02 | 0.03±0.01 | 0.07±0.01 | 0.10±0.08 | 0.02±0.01 | 0.03±0.02 | 0.08±0.03 | 0.46±0.25 |
| Flavobacteriales | 0.02±0.03 | 0.01±0.00 | 0.02±0.01 | 0.03±0.01 | 0.01±0.01 | 0.02±0.01 | 0.02±0.03 | 0.11±0.23 | 0.02±0.00 | 0.01±0.01 | 0.01±0.00 | 0.16±0.27 |
| Bacillales | 0.07±0.09 | 0.10±0.05 | 0.11±0.16 | 0.07±0.02 | 0.04±0.02 | 0.21±0.11 | 0.09±0.03 | 0.10±0.09 | 0.04±0.01 | 0.26±0.19 | 0.07±0.02 | 0.05±0.02 |
| Nevskiales | 0.02±0.03 | 0.00±0.00 | 0.01±0.01 | 0.07±0.07 | 0.00±0.01 | 0.00±0.00 | 0.00±0.00 | 0.09±0.17 | 0.01±0.02 | 0.00±0.00 | 0.01±0.00 | 0.00±0.00 |
| Mycobacteriales | 0.06±0.05 | 0.01±0.01 | 0.04±0.02 | 0.04±0.02 | 0.13±0.14 | 0.03±0.02 | 0.06±0.04 | 0.07±0.11 | 0.06±0.02 | 0.04±0.03 | 0.07±0.05 | 0.03±0.02 |
| Pseudomonadales | 0.06±0.06 | 0.04±0.02 | 0.07±0.07 | 0.03±0.02 | 0.04±0.03 | 0.06±0.03 | 0.06±0.06 | 0.08±0.11 | 0.21±0.11 | 0.08 ± 0.11 | 0.04±0.02 | 0.02±0.00 |
| Burkholderiales | 0.06±0.05 | 0.04±0.01 | 0.06±0.01 | 0.19±0.04 | 0.05±0.02 | 0.10±0.05 | 0.08±0.06 | 0.12±0.14 | 0.08±0.05 | 0.04±0.01 | 0.07±0.04 | 0.04±0.03 |
| Enterobacterales | 0.03±0.02 | 0.04±0.00 | 0.08±0.05 | 0.11±0.05 | 0.07±0.05 | 0.08±0.06 | 0.05±0.03 | 0.08±0.10 | 0.03±0.01 | 0.04±0.07 | 0.08±0.07 | 0.02±0.01 |
| Rhodospirillales | 0.01±0.00 | 0.01±0.00 | 0.01±0.00 | 0.07±0.09 | 0.01±0.00 | 0.01±0.01 | 0.01±0.01 | 0.01±0.01 | 0.01±0.01 | 0.01±0.01 | 0.01±0.01 | 0.01±0.01 |
| Oceanospirillales | 0.00±0.00 | 0.00±0.00 | 0.01±0.02 | 0.01±0.01 | 0.01±0.02 | 0.00±0.00 | 0.00±0.00 | 0.00±0.00 | 0.00±0.00 | 0.00±0.00 | 0.05±0.09 | 0.01±0.01 |
| Rhodobacterales | 0.01±0.00 | 0.01±0.03 | 0.05±0.08 | 0.00±0.00 | 0.02±0.02 | 0.01±0.02 | 0.02±0.01 | 0.01±0.00 | 0.01±0.00 | 0.00±0.00 | 0.01±0.01 | 0.01±0.00 |
| Poales | 0.01±0.01 | 0.13±0.05 | 0.00±0.00 | 0.00±0.00 | 0.00±0.00 | 0.00±0.00 | 0.00±0.00 | 0.00±0.00 | 0.01±0.00 | 0.05±0.03 | 0.00±0.00 | 0.00±0.00 |
| Bacteroidales | 0.00±0.00 | 0.01±0.00 | 0.00±0.00 | 0.00±0.00 | 0.00±0.00 | 0.03±0.04 | 0.02±0.02 | 0.00±0.00 | 0.04±0.07 | 0.00±0.00 | 0.01±0.00 | 0.00±0.00 |
| Streptosporangiales | 0.03±0.03 | 0.00±0.00 | 0.00±0.00 | 0.00±0.00 | 0.01±0.01 | 0.00±0.01 | 0.00±0.00 | 0.00±0.00 | 0.09±0.06 | 0.01±0.01 | 0.00±0.00 | 0.00±0.00 |
| Pseudonocardiales | 0.00±0.00 | 0.00±0.00 | 0.01±0.00 | 0.00±0.00 | 0.01±0.00 | 0.00±0.00 | 0.04±0.06 | 0.00±0.00 | 0.00±0.00 | 0.00±0.00 | 0.02±0.02 | 0.00±0.00 |
| Lactobacillales | 0.01±0.00 | 0.01±0.01 | 0.01±0.01 | 0.01±0.01 | 0.01±0.01 | 0.03±0.02 | 0.02±0.01 | 0.01±0.01 | 0.01±0.01 | 0.02±0.00 | 0.04±0.05 | 0.01±0.01 |
| Nitrosomonadales | 0.00±0.00 | 0.00±0.00 | 0.01±0.02 | 0.05±0.05 | 0.01±0.01 | 0.00±0.00 | 0.01±0.00 | 0.01±0.02 | 0.01±0.01 | 0.00±0.00 | 0.01±0.01 | 0.00±0.00 |
| Others | 0.19±0.14 | 0.53±0.06 | 0.33±0.07 | 0.17±0.03 | 0.24±0.11 | 0.33±0.10 | 0.36±0.05 | 0.15±0.09 | 0.27±0.06 | 0.37±0.08 | 0.35±0.07 | 0.14±0.08 |
